# Compared efficacy of clopidogrel and ticagrelor in treating acute coronary syndrome: a meta-analysis

**DOI:** 10.1186/s12872-018-0948-4

**Published:** 2018-11-29

**Authors:** Dong Wang, Xiao-Hong Yang, Ji-Dong Zhang, Rui-Bin Li, Min Jia, Xiao-Ran Cui

**Affiliations:** Department of Sixth Cardiovascular Medicine, Second Hospital of Hebei Medical University, No. 309 North Zhonghua Street, Shijiazhuang, 05400 Hebei China

**Keywords:** Ticagrelor, Clopidogrel, Acute coronary syndrome, Meta-analysis

## Abstract

**Background & Aims:**

Ticagrelor has been acknowledged as a new oral antagonist of P2Y12-adenosine diphosphate receptor, as a strategy with more rapid onset as well as more significant platelet inhibition function in acute coronary syndrome (ACS) patients. The clinical benefit of ticagrelor compared with clopidogrel remains controversial. The current meta-analysis was conducted to better evaluate the role of ticagrelor in comparison of clopidogrel in treating ACS patients.

**Methods:**

The publications involving the safety as well as the efficacy of clopidogrel versus ticagrelor were screened and identified updated to June 2018. After rigorous review, eligible randomized controlled trials (RCTs) were extracted and propensity score matching (PSM) analysis was conducted. To analyze the summary odds ratios (ORs) of the endpoints of interest, we applied Meta-analysis Revman 5.3 software.

**Results:**

There were a total of 10 studies that met our inclusion criteria, of which the risk of bleeding rate (*P* = 0.43), MI (*P* = 0.14), and stroke (*P* = 0.70) had no association with significant differences between patients receiving ticagrelor or clopidogrel. Nonetheless, higher rate of dyspnea was observed in ticagrelor group (OR = 1.87, 95% CI: 1.70–2.05, *P*<0.00001 = .

**Conclusions:**

Our present findings suggest similar efficacy and safety profiles for clopidogrel and ticagrelor Ticagrelor should be considered as a valuable option to reduce the risk of bleeding, MI and stroke, whereas potentially increases the incidence of dyspnea. Given the metabolic process, ticagrelor may be a valid and even more potent antiplatelet drug than clopidogrel, as an alternative strategy in treating patients with clopidogrel intolerance or resistance.

## Introduction

Acute coronary syndrome (ACS) is a series of urgent clinical syndromes in the coronary arteries because of decreased blood flow. It has been acknowledged that occurrence as well as the development of ACS has a strong link to platelet aggregation. Therefore, standard treatment has been established with the use of dual antiplatelet therapy (DAPT) with P2Y12 receptor inhibitor and aspirin for ACS patients regardless previous treatments, such as medical management or percutaneous coronary intervention (PCI) [1, 2].

Clopidogrel, a P2Y12 receptor antagonist as a valid antiplatelet drug for patients ACS patients, has been extensively used worldwide for over a decade. As a prodrug, it often requires hepatic conversion and leads to onset delay of metabolites with a wide variability of platelet inhibition between individuals, and more than one-third of them display minimal platelet inhibition or “clopidogrel non responders” [3–5]. Moreover, the high bleeding risk, stent thrombosis, myocardial infarction (MI) and poor response of patients with the use of clopidogrel show the limitation of its efficacy [6–8]. Hence, slow onset and low potency of platelet inhibition of clopidogrel has been found based on previous publications [9].

Ticagrelor is a direct-acting oral antagonist of P2Y12-adenosine diphosphate (ADP) receptor blocker with reversibility and without catabolite activation, which can have a substantial impact on faster and greater platelet inhibition than clopidogrel [10, 11]. Ticagrelor has more remarkable beneficial outcomes in reversible long-term P2Y12 inhibition than clopidogrel in the total death, cardiovascular prevention, stent thrombosis as well as myocardial infarction without increasing the major bleeding rates in a wide ACS patient population with timely intervention, according to the Phase III PLATO (Platelet Inhibition and Patient Outcomes) trial [12]. Thus, several clinical management guidelines suggested that ticagrelor could be a valid strategy and associated with superior effects over clopidogrel for P2Y12 inhibition in ACS patients [13, 14].

Earlier studies have been published for the assessment of safety and efficacy of ticagrelor versus clopidogrel in ACS patients Nevertheless, given the differences of genetic backgrounds, comorbidities, disease patterns, and demographics, patients tend to show various prognostic results with uncertain bleeding risk [15, 16].

Therefore, attempts have been made in the present study in order to offer conclusive clinical evidence on the controversial results through this meta-analysis that evaluates the safety and efficacy profile of ticagrelor versus clopidogrel in ACS patients.

## Methods and materials

### Search strategy

An electronic search of literature using Embase, PubMed, and the Cochrane Library was conducted by two reviewers separately up to June 2018. All publications with the following keywords were included: “Ticagrelor”; “Clopidogrel” and “Acute coronary syndrome”. We also applied the associated Medical Subject Heading (MeSH) terms. References from relevant studies and review literatures were further searched to confirm retrieval of all possible pertinent trials.

### Eligibility criteria

Qualified Studies were assessed based on the following criteria [1]: the studies are designed as random control trials (RCTs) and propensity score matching (PSM) control trials comparing clopidogrel versus ticagrelor [2]; inclusion of patients with ACS [3]; the incidence of vascular effects as well as major adverse cardiovascular were considered as the primary efficacy end point, which was also defined as the composite events of stroke, myocardial infarction(MI), bleeding and dyspnea.

### Quality assessment

The quality of retrieved studies was rated and collected separately by two studiers (Dong Wang and Xiao-Hong Yang). And the quality of observational studies was under assessment with the use of Newcastle- Ottawa Quality Assessment Scale [17]. RCTs were graded using the Jadad scale [18].

### Data extraction

Data from each included study were checked and collected for consistency by two investigators. Any arising disagreements were settled through discussion to reach general consensus. The main categories from each of the eligible studies were included on the basis of the following parameters: family name of first author, year of publication, study design, dose and method of drugs, number of patients, follow-up time, and clinical outcome.

### Statistical analysis

The statistical analyses were carefully conducted through the use of Review Manager version 5.3 software (Revman; The Cochrane collaboration Oxford, United Kingdom). We used the inverse variance method for the calculation of endpoints of interest based on ORs with their 95% confidence intervals (CIs); the individual estimate of the OR was weighted through endpoint outcomes across each study.

The heterogeneity across studies was examined through the I^2^ statistic to evaluate the sensitivity [19], describing as follows respectively: low, I^2^<50%; moderate or high, I^2^ ≥ 50% [20]. We applied the fixed-effect models when low heterogeneity showed in studies. In other cases, we used the random effects model. Studies with a *P* value less than 0.05 was thought to have statistical significance.

## Results

### Searched outcomes and general features of the trials

**Fig. 1:**
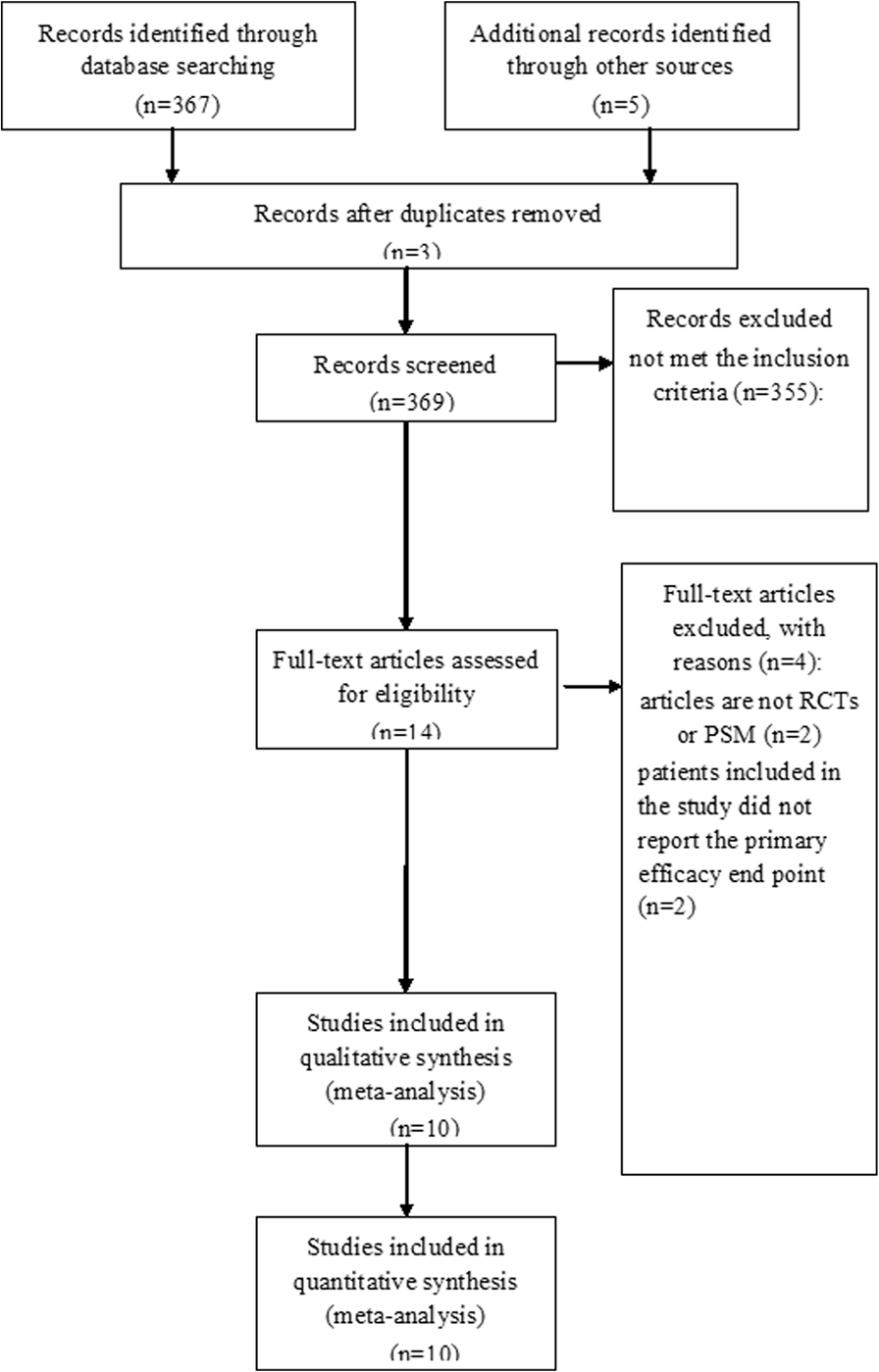
PRISMA flow chart of selection process to identify eligible studies

Electronic search of literatures resulted in a total number of 369 publications originally. On the basis of the abovementioned criteria, assessment of detail was carried out and 14 publications were included, whereas some articles were excluded due to the lack of outcomes of two approaches. Hence, there were a total number of 10 studies with eligibility [12, 21–29]. Figure 1 revealed the detailed search process.

The abovementioned studies were based on evidence with moderate to high quality. Table 1 described the major characteristics of the qualified studies in more detail.

**Table 1:** Major characteristics of the included studies

Study	Publication year	Study design	No. Patients		Drug Dose		Follow-up, mo	Clinical Outcome
			Ticagrelor	Clopidogrel	Ticagrelor, mg.q.d	Clopidogrel, mg.q.d		
Cannon	2007	RCT	6732	6676	90 mg.bid	LD: 300 MD: 75	3	1、2、3、4
Lars Wallentin	2009	RCT	9333	9291	LD: 180 mg.bid MD: 90 mg.bid	LD: 300–600 MD: 75	12	1、2、3、4
Cannon	2010	RCT	663	327	LD: 180 mg.bid MD: 90 mg.bid	LD: 300 MD: 75	12	1、2、3
Laurent Bonello	2014	RCT	30	30	LD: 180 mg.bid MD: 90 mg.bid	LD: 600 MD: 75	N	4
Y. Hiasa	2014	RCT	93	46	90/45 mg.bid	75	3	3
Shinya Goto	2015	RCT	401	400	LD: 180 mg.bid MD: 90 mg.bid	LD: 300 MD: 75	12	1、2、3
Huidong Wang	2016	RCT	100	100	LD: 180 mg.bid MD: 90 mg.bid	LD: 300 MD: 75	12	1、2、3
Ran Xiong	2015	RCT	112	112	LD: 180 mg.bid MD: 90 mg.bid	LD: 600 MD: 150	12	1
I-Chih Chen	2016	PSM	224	224	/	/	12	1、2、3、4
Cheng-Han Lee	2018	PSM	2389	19,112	LD: 180 mg.bid MD: 90 mg.bid	LD: 300–600 MD: 75	18	1、2、3

*RCT* random control trial, *PSM* propensity score matching, *LD* loading dose, *MD* maintenance dose

Outcome [1]: MI [2]; stroke [3]; TIMI-defined bleeding [4]; Dyspnea

### Clinical and methodological heterogeneity

#### Pooled analysis of the risk of bleeding

**Fig. 2:**
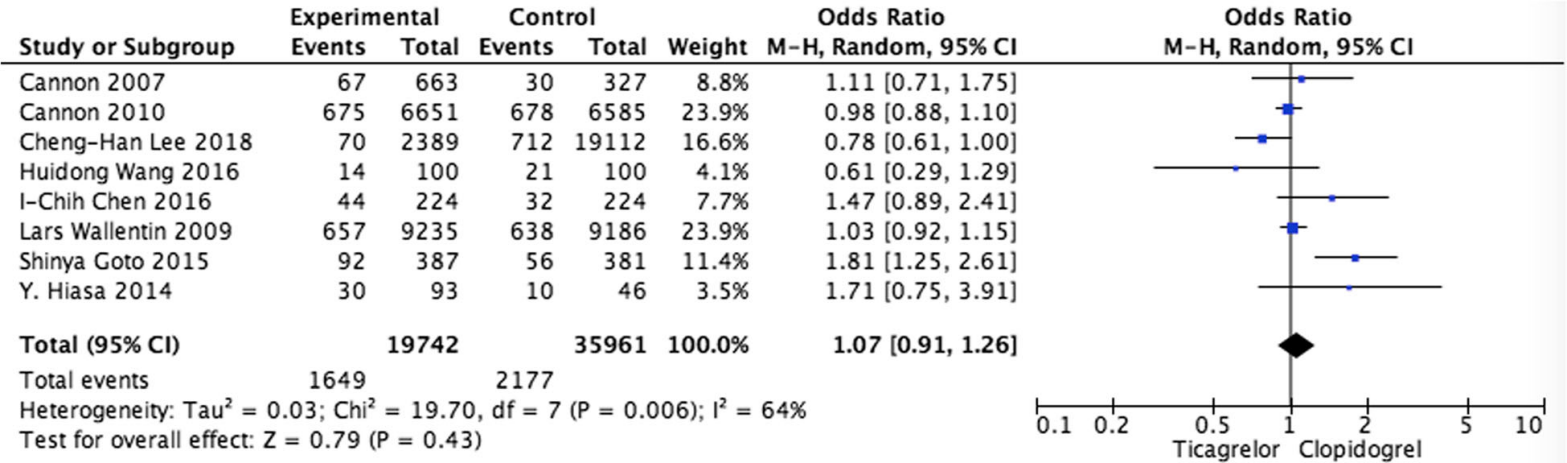
Pooled analysis of the risk of bleeding between ticagrelor and clopidogrel

Pooled data from 8 studies showed no differences in the risk of bleeding (OR = 1.07, 95%CI: 0.91–1.26, *P* = 0.43) when comparing ticagrelor and clopidogrel group (Fig. 2).

**Fig. 3:**
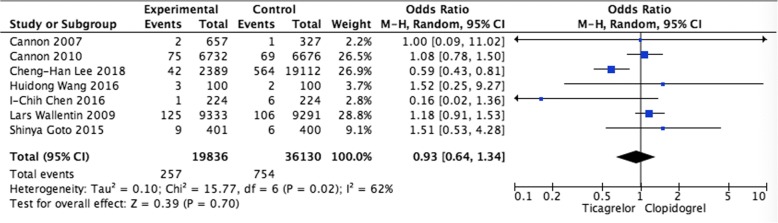
Pooled analysis of stroke between ticagrelor and clopidogrel

Pooled analysis of stroke Stroke rate was available for 7 trials. No significant differences were observed when comparing ticagrelor and clopidogrel (OR = 0.93, 95% CI: 0.64–1.34, *P* = 0.70) (Fig. 3).

#### Pooled analysis of MI

**Fig. 4:**
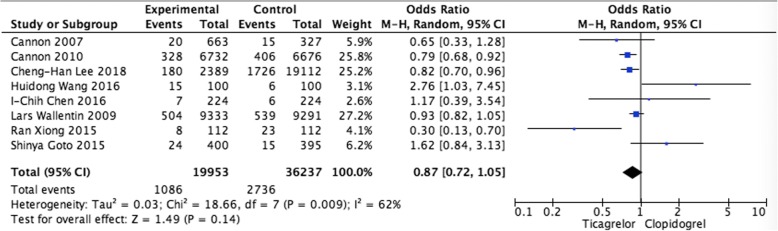
Pooled analysis of MI between ticagrelor and clopidogrel

In the analysis of MI in patients who were treated with ticagrelor or clopidogrel, eight studies were included. Pooled data revealed that ticagrelor was not associated with higher trend of rate than clopidogrel, with the pooled OR being 0.87 (95% CI 0.72–1.05, *P* = 0.14) (Fig. 4).

**Fig. 5:**
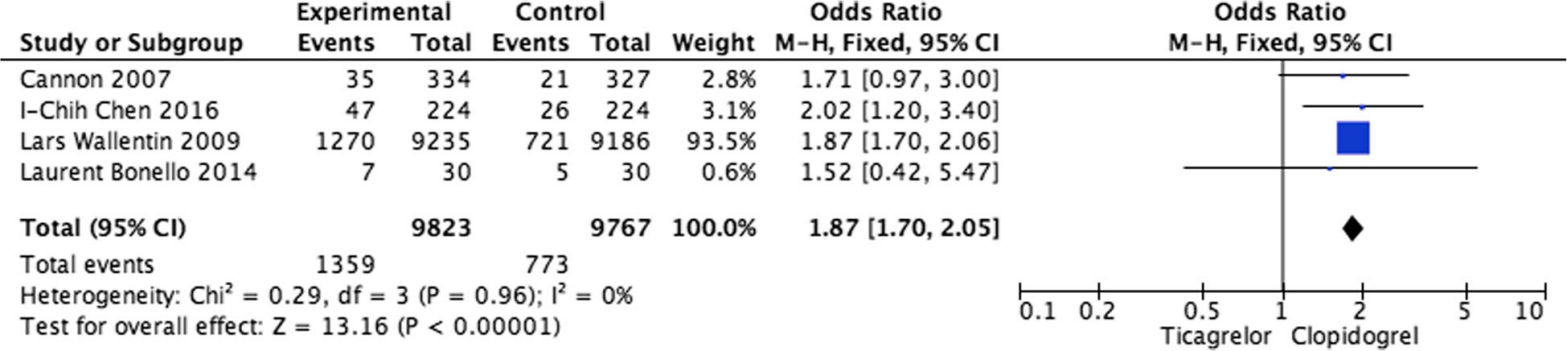
Pooled analysis of dyspnea events between ticagrelor and clopidogrel

Pooled analysis of dyspnea events The incidences of dyspnea events in patients with ACS were available for four studies (Fig. 5). The pooling analysis revealed that ticagrelor was linked to a higher rate of dyspnea (OR = 1.87, 95% CI: 1.70–2.05, *P*<0.00001).

## Discussion

Dual antiplatelet therapy, usually accompanied with a P2Y12 receptor antagonist and aspirin, is generally acknowledged as a vital approach in treating ACS patients, partly because of the increased occurrence of thrombogenesis. Dual antiplatelet therapy has been also regarded as a standard therapy especially after PCI according to several clinical guidelines [14, 30].

Clopidogrel, a P2Y12 receptor antagonist, has been generally utilized with aspirin as prescribed antiplatelet agents in an attempt to decrease the MI risk and stent thrombosis in patients with acute coronary syndromes with or without ST elevation [22]. However, clopidogrel requires 2-step hepatic metabolism as an inactive pro-drug that has a strong link to delayed onset and various responses [31, 32].

Ticagrelor is a direct-acting oral antagonist of P2Y12-receptor antagonist with reversibility and without catabolite activation, which can have a substantial impact on faster with consistent greater platelet inhibition than clopidogrel [10, 12].

However, previous trial supported by some studiers demonstrated no remarkable difference in terms of the bleeding rate with the use of ticagrelor in comparison of clopidogrel. Additionally, results of ventricular pauses on Holter monitoring as well as the dose-related episodes of dyspnea were found with high occurrence when using ticagrelor [12]. We conducted the study to evaluate the function of ticagrelor in terms of its superior effect to clopidogrel for ACS patients.

The present analysis showed no statistical reduction of bleeding incidence, MI as well as stroke in ticagrelor in comparison of clopidogrel. Nonetheless, dyspnea was more common in the ticagrelor group.

Although susceptibility of higher bleeding risk was affected by platelet inhibition, controversy existed regarding to the link of bleeding risk and platelet inhibition level, of which the risk of major bleeding based on platelet inhibition level has not been estimated [33, 34]. As shown in human race, there is variation in the drug response with different populations. Comparing with non-Asian patients, Asian patients tend to be more susceptive with high bleeding risk in terms of the lower body weight, different genetic background, disease patterns as well as comorbidities [35]. Higher bleeding risk in Asian population has gained interests with standard doses of new P2Y12 inhibitors, especially in East Asian patients [36]. Furthermore, on basis of clinical experience and evidence, there may be a dissimilarity between the Chinese and Japanese patients [37, 38]. It is always important to take different genetic predisposition into consideration in order to perform proper antiplatelet therapy if clopidogrel is applied as a control. Compared with clopidogrel, ticagrelor is less likely to be influenced by CYP2C19 polymorphism [39]. The role of platelet inhibition in ticagrelor may gain popularity among Asian patients who are prone to have higher prevalence of loss of CYP2C19 function polymorphism, which may be associated with the remarkable decrease of ischemic events [40].

Increased risk of cardiovascular events usually leads to bleeding [41]. However, the risk factors of subsequent cardiovascular events were associated with major bleeding with differences in degree between Asian and non-Asian patient individuals. Debates exists concerning the reason for the susceptibility of Asian patients to subsequent cardiovascular events. Asian patients who suffer numerically higher subsequent ischemic events are those who are associated with major bleeding than without bleeding [40]. In addition, another strong predictor of adverse prognosis after ACS is the age of patients [42, 43]; ACS patients with older age tend to be accompanied with drug-related bleeding complications and increased risk of CV death. Previous clinical prognosis of older age with ACS is often further complicated by more common comorbidity [44, 45]. The abovementioned findings need further evaluation to offer strong evidence.

Dyspnea is another pivotal parameter as adverse effect in addition to bleeding. In our study, ticagrelor exerted increased dyspnea incidence in comparison of clopidogrel, which mainly due to its function in increasing the inhibition of P2Y12 on sensory neurons and the endogenous adenosine concentration [46–48].

The main strength of our study is the use of a well-maintained and updated database including studies that were designed as random control trials (RCTs) and propensity score matching (PSM) control trials. Nevertheless, potential bias exists by the intrinsic retrospective study and the imbalance in baseline demographics and clinical characteristics, which may impact the comparison of relevant outcomes. More large-scale studies with greater statistical significance are warranted for the confirmation of the safety as well as efficacy profile of ticagrelor and clopidogrel.

## Conclusion

In summary, we present a meta-analysis with evidence-based data comparing ticagrelor and clopidogrel in treating ACS patients. Aggregated results showed no increase in major bleeding rate, MI and stroke with the use of ticagrelor except for dyspnea rate. However, considering ticagrelor is less likely to be influenced by metabolic activation and various drug action between individuals, it has potential to be a valid, alternative antiplatelet drug in comparison of clopidogrel. Hence, ticagrelor may be a valid and even more potent antiplatelet drug than clopidogrel, especially as an alternative strategy in treating patients with clopidogrel intolerance or resistance.
